# Cardiac myxoma and cerebral vasculitis: Is there a link?

**DOI:** 10.1186/s43044-024-00488-0

**Published:** 2024-05-23

**Authors:** Samah El-Mhadi, Belghait El Hajjaj, Asmae Benatmane, Mariam El Harrak, Sara Ahchouch, Abderrahim Elktaibi, Fouad Nya, Najat Mouine, Aatif Benyass

**Affiliations:** 1 Department of Clinical Cardiology, Mohammed V Military Hospital, Rabat, Morocco; 2 Department of Cardiovascular Surgery, Mohammed V Military Hospital, Rabat, Morocco; 3 Department of Pathology, Mohammed V Military Hospital, Rabat, Morocco

**Keywords:** Cardiac myxoma, Acute ischemic stroke, Cerebral vasculitis, Open-heart surgery

## Abstract

**Background:**

Cardiac myxomas present a diagnostic challenge due to their ability to mimic various cardiovascular and systemic conditions. Timely identification is crucial for implementing surgical intervention and averting life-threatening complications.

**Case presentation:**

We reported the case of a 49-year-old male patient who presented sudden legs weakness and slurred speech and was admitted 10 h later in emergency department. Physical examination was significant for paraparesis and paraphasia. Cardiac and carotid auscultation was normal. CT brain revealed multiple acute ischemic strokes and MRA was suggestive of cerebral vasculitis. As pre-therapy assessment, the EKG revealed no electrical abnormalities and the chest X-ray showed signs of left atrial enlargement. Transthoracic and transesophageal echocardiography showed a left atrial mass attached to the interatrial septum, measuring 9*5*4 cm and extending into the left ventricular cavity during diastole, which suggested the diagnosis of left atrial myxoma. The patient was referred for open-heart surgery and histopathological examination confirmed the diagnosis of myxoma. The patient weaned off from cardiopulmonary bypass and the postoperative period was uneventful.

**Conclusion:**

We reported an interesting case with an unusual and misleading neurological presentation of a cardiac myxoma. The unpredictability of serious complications occurrence must awaken our medical flair, for an early diagnosis among a long list of differentials.

## Background

Cardiac myxomas present a considerable diagnostic challenge in medical practice. These tumors, primarily originating from the endocardium, can masquerade as various cardiovascular and systemic conditions, complicating their timely diagnosis and management [1]. Literature underscores the importance of a high index of suspicion for cardiac myxomas, particularly in patients with unexplained cardiac or systemic symptoms, in order to provide surgical treatment and prevent life-threatening complications [1].

## Case presentation

We reported the case of a 49-year-old male patient with no cardiovascular risk factors or medical history, who experienced sudden onset of leg weakness and slurred speech. He presented to the emergency department 10 h later, with a heart rate of 100 bpm and blood pressure of 130/85 mmHg in both arms. Physical examination revealed paraparesis and paraphasia. Cardiac and carotid auscultation was normal.

**Fig. 1:**
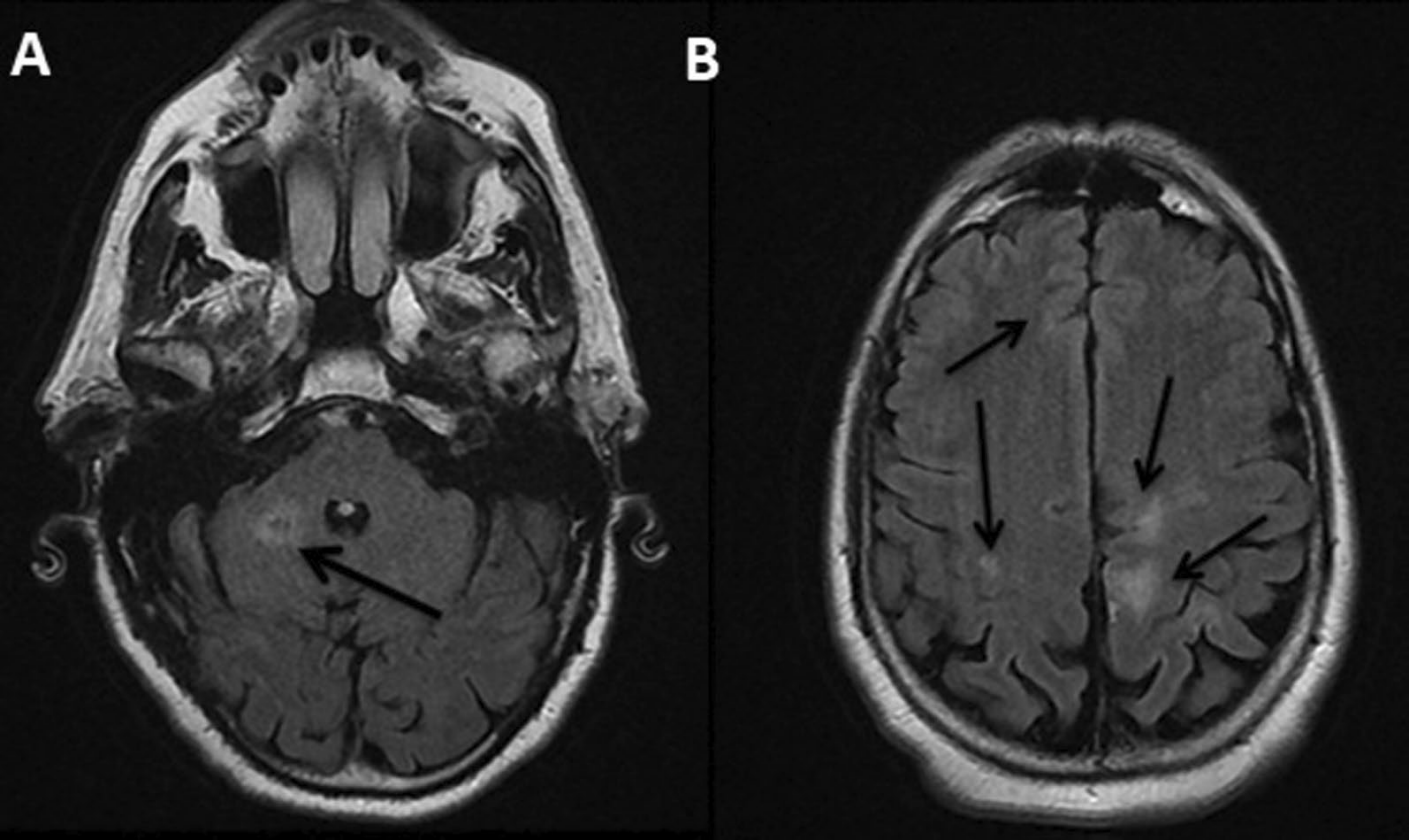
**A** + **B** Axial views of brain MRI showing frontal, occipital and cerebellar white matter signal abnormalities

CT brain showed multiple acute ischemic strokes. MRA revealed frontal, occipital and cerebellar white matter signal abnormalities suggestive of cerebral vasculitis (Fig. 1). The patient was subsequently transferred to the internal medicine department for further investigations and vasculitis treatment.

**Fig. 2:**
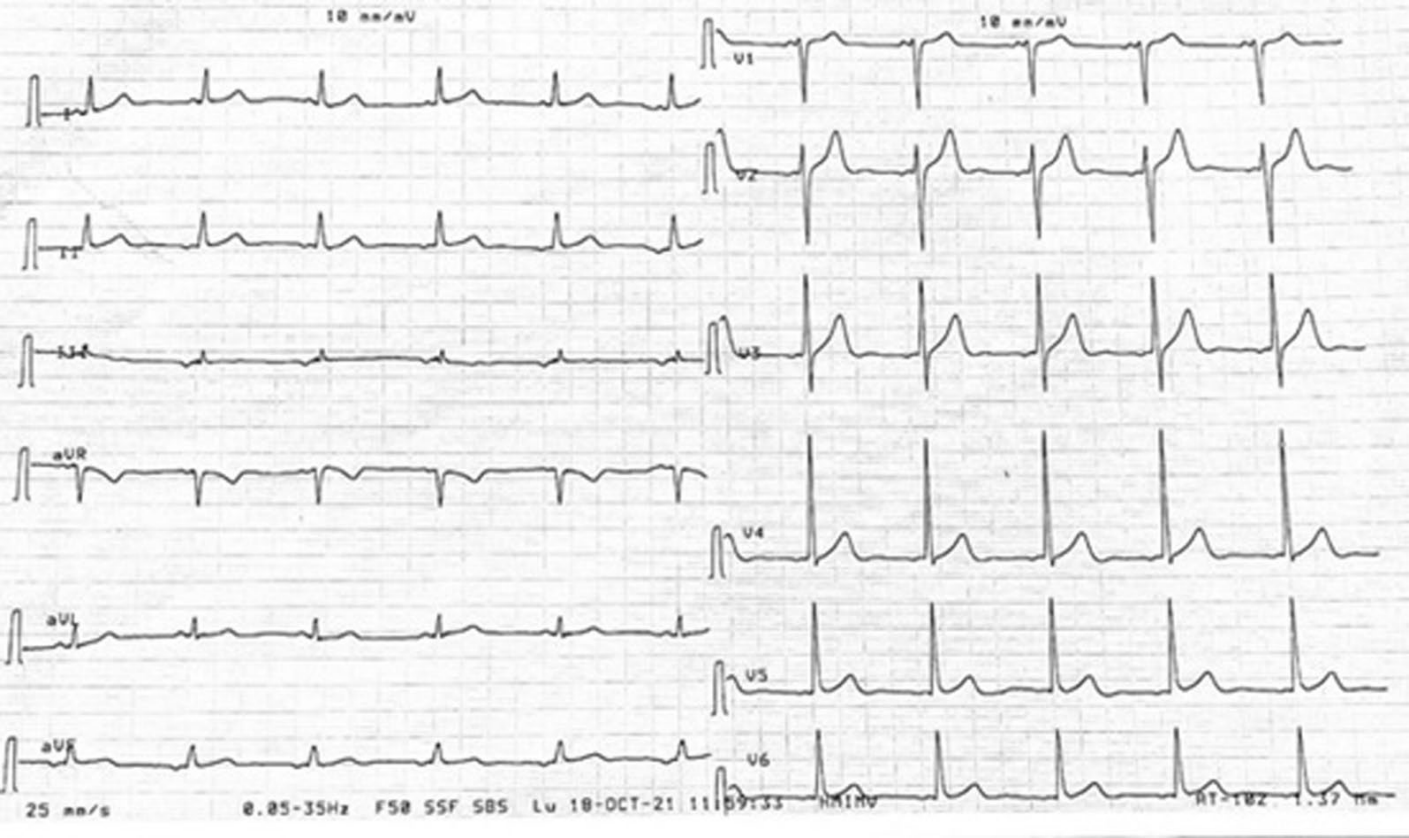
EKG showing coronary sinus rythm

**Fig. 3:**
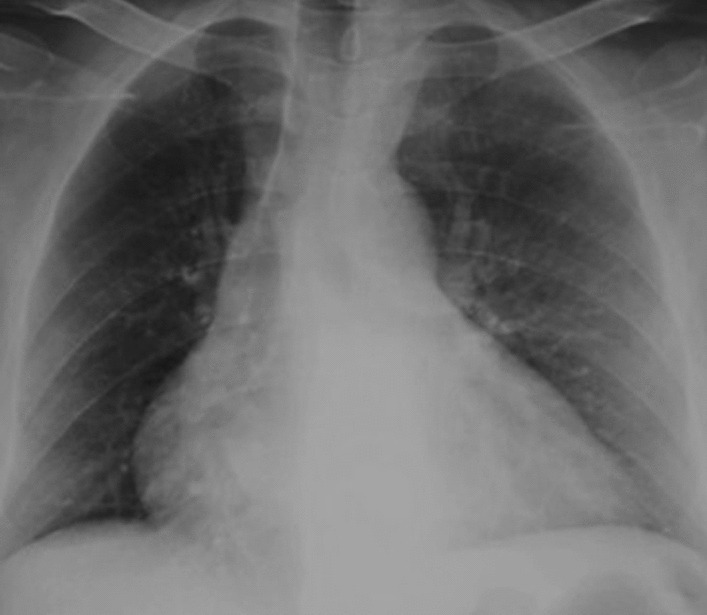
Chest X-ray showing signs of left atrial enlargement

**Fig. 4:**
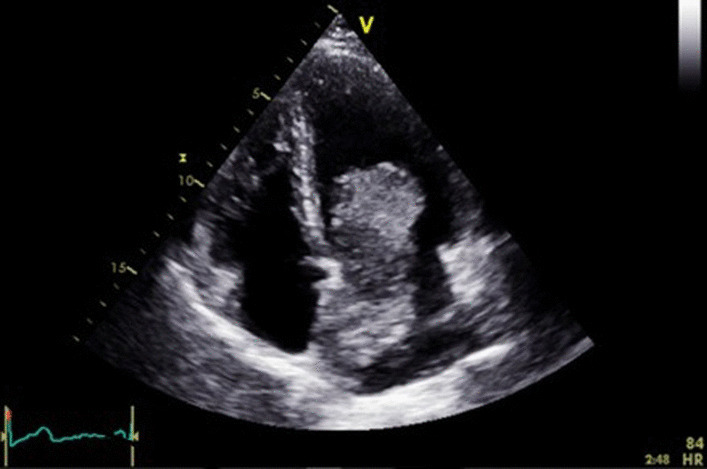
Cine four-chamber view of TTE revealing a huge left atrial myxoma

**Fig. 5:**
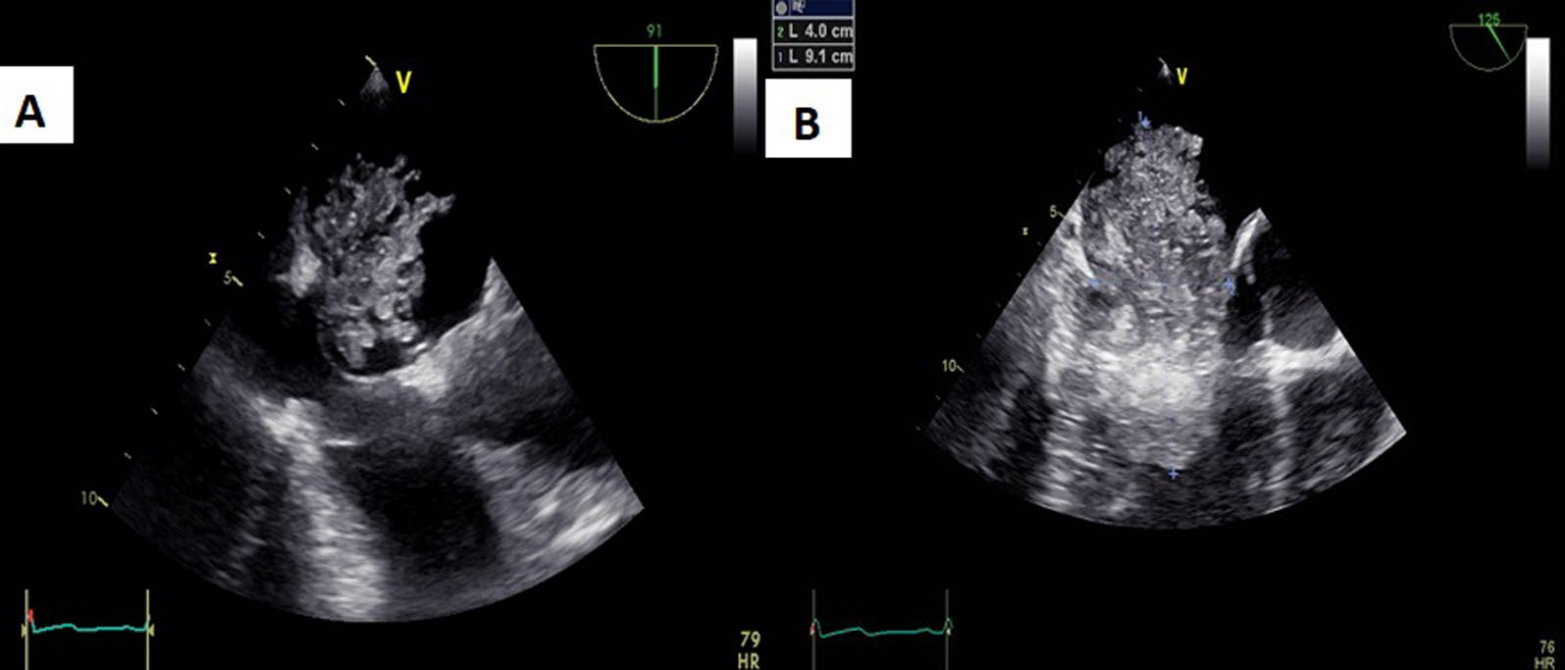
**A** + **B** Cine views of TOE showing the dimensions of myxoma and its attachment to the interatrial septum

Pre-therapy assessments included an EKG which revealed a coronary sinus rhythm without electrical abnormalities (Fig. 2), a chest X-ray which showed cardiomegaly with a double density sign and splaying of the carina indicative of left atrial enlargement (Fig. 3), and a transthoracic echocardiography (TTE) which revealed a mobile and heterogeneous mass measuring 9*5*4 cm in the left atrium, extending into the left ventricular cavity during diastole without significant inflow obstruction across the mitral valve (Fig. 4). Transesophageal echocardiography (TOE) confirmed that the mass was attached to the interatrial septum at the site of the fossa ovalis, consistent with a diagnosis of left atrial myxoma (Fig. 5).

The patient underwent preoperative supra-aortic trunk echo-Doppler and coronary calcium scan which were unremarkable.

Laboratory examinations revealed microcytic and hypochromic anemia (hemoglobin: 10 g/dL) and elevated inflammatory biomarkers (C-reactive protein: 39 mg/L, ferritinemia: 520 ng/mL), with no hydro-electrolyte disorder and normal liver and kidney functions. Serological tests for syphilis, HIV, VHB and VHC and autoimmune screening were negative.

**Fig. 6:**
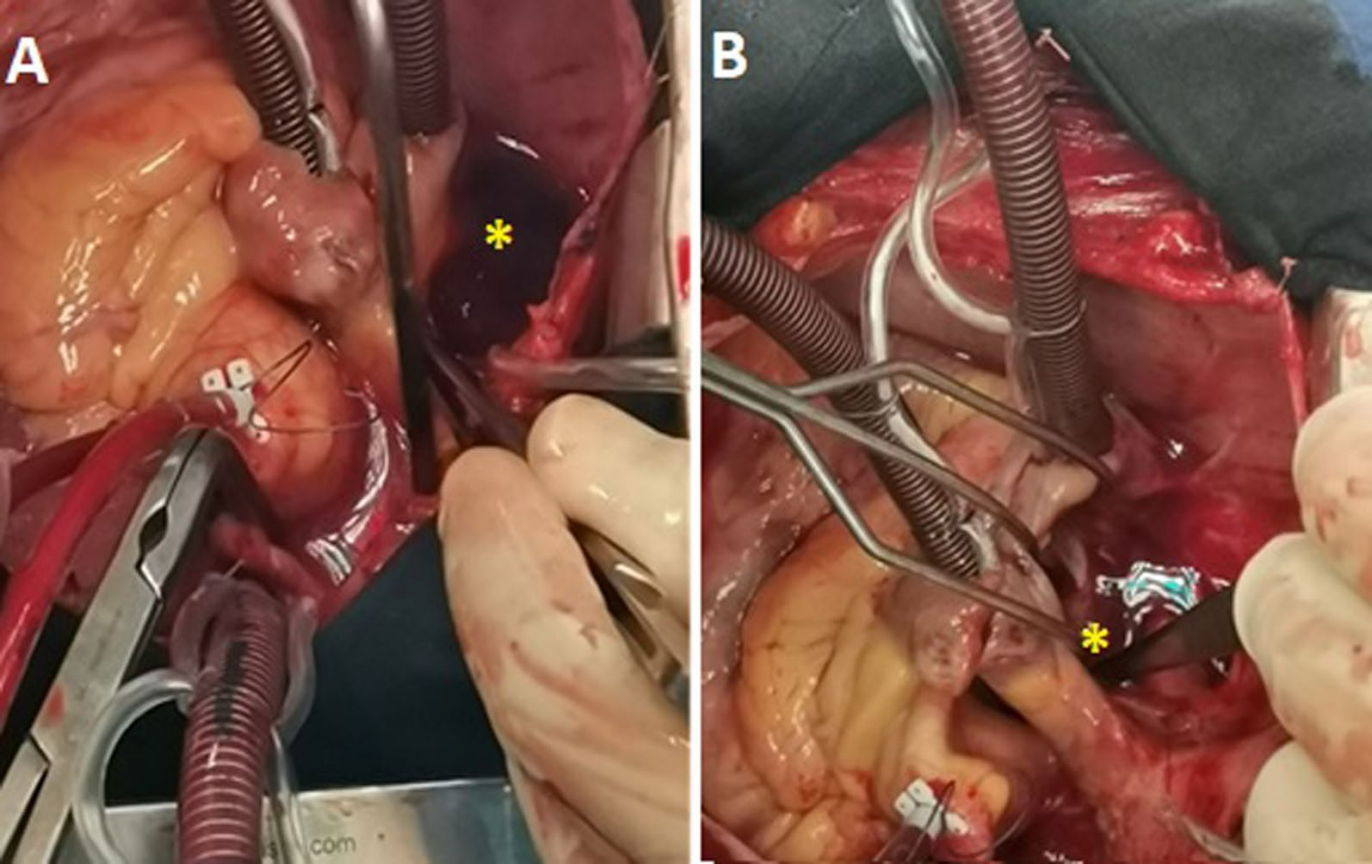
**A** + **B** Intraoperative photographs showing the left atrial myxoma (*)

The patient underwent open-heart surgery. An aorto-bicaval cannulation and a cardiopulmonary bypass were established. After left atriotomy, a giant tumor arising from the interatrial septum was resected, with cauterization of its implantation base in order to prevent local recurrence. The left atrium was closed, and the aortic cross-clamp was removed. The surgical intervention lasted 50 min (Fig. 6).

**Fig. 7:**
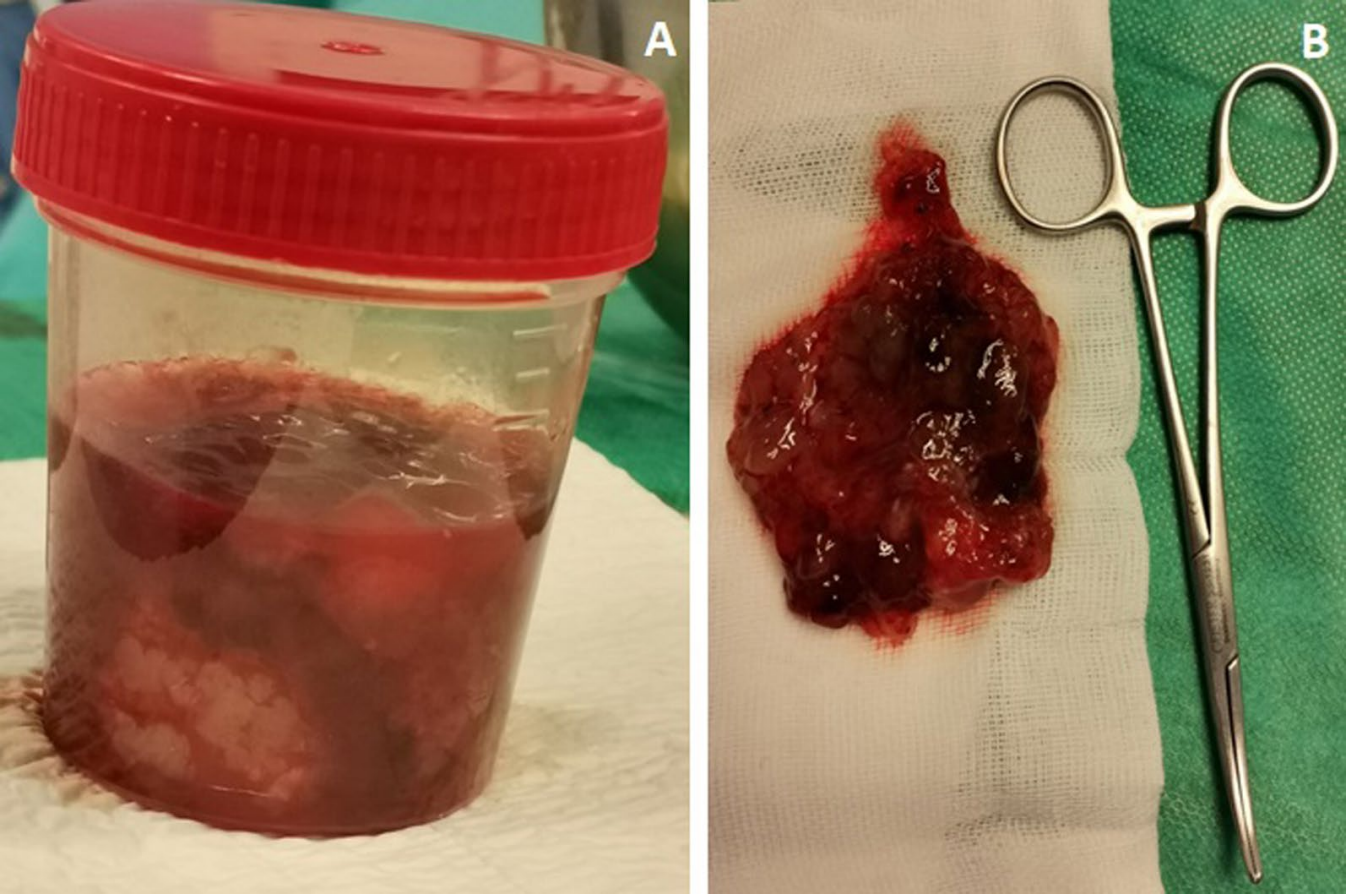
**A** + **B** Macroscopical aspects of the resected specimen

**Fig. 8:**
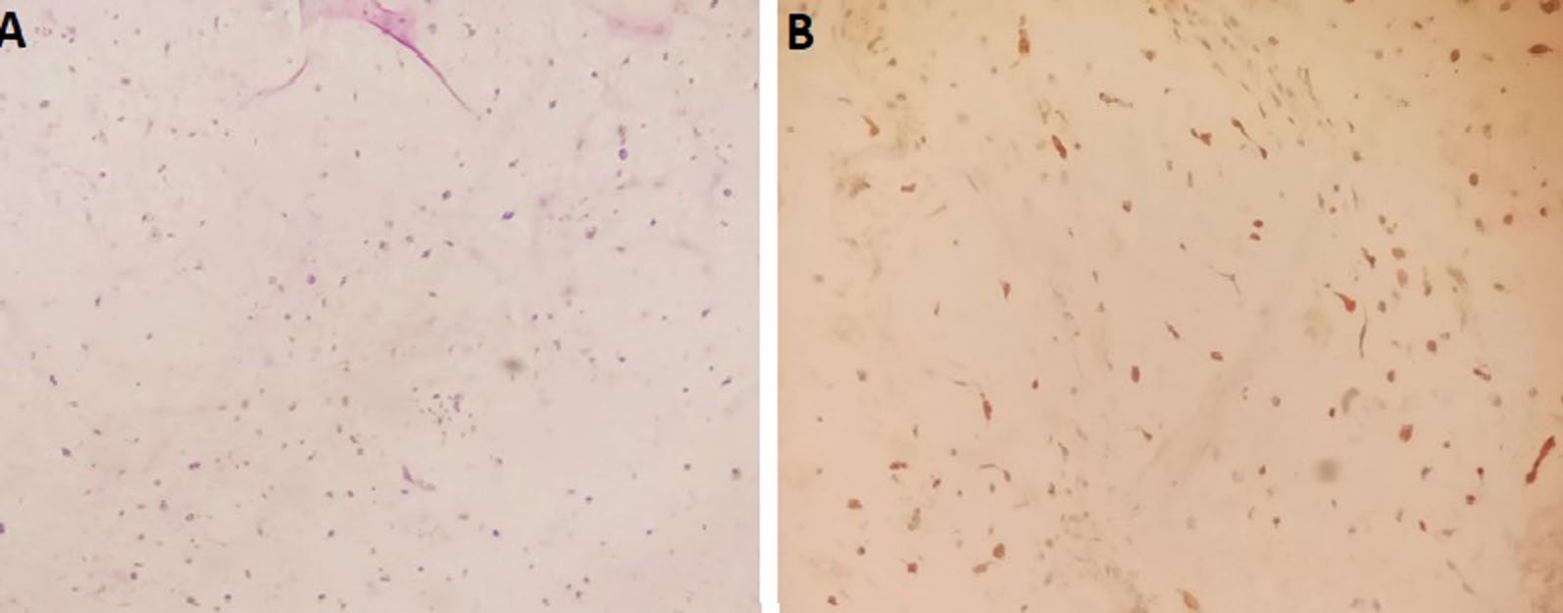
**A** Low Power View of atrial myxoma showing the tumor cells within a myxoid stroma (Haematoxylin and Eosin stained sections); **B** Calretinin Immunoreactivity shown in myxoma’s cells

Macroscopically, the excised mass measured 9*4*4 cm and was soft-mulberry-shaped and friable, with gelatinous consistency and punctate hemorrhage on the outer surface (Fig. 7). Histopathological examination showed marked loose myxoid stroma with scattered spindle cells, but no mitotic figures or malignant features, confirming the diagnosis of myxoma (Fig. 8).

Postoperative TOE did not reveal an interatrial shunt. The patient was monitored in the ICU for 24 h. The postoperative period was uneventful. He was discharged home with long-term echocardiographic follow-up recommended. The patient reported complete neurological recovery one month later.

## Discussion

Primary cardiac tumors are rare entities in medicine, with studies indicating an incidence ranging from 0.001% to 0.3% in non-selected populations.

Three-quarters of these tumors are benign, and 30% of benign cardiac tumors are represented by myxomas [2].

Myxomas are typically diagnosed between the ages of 30 and 60 years. They mostly develop in the left atrium, specifically from the interatrial septum at the fossa ovalis, compared to right atrial and ventricular locations [2].

Clinical features of cardiac myxomas are determined by their size, location, consistency, and mobility. Constitutional symptoms attributed to myxomas, such as fever, weight loss, and arthralgia, suggest systemic inflammatory responses that could extend to the vascular endothelium [3].

The understanding of cardiac myxomas extends beyond their local impact within the heart, with emerging considerations for systemic manifestations, including their potential association with cerebral vasculitis [4].

Studies have highlighted that cardiac myxomas can lead to ischemic acute cerebral strokes through embolic phenomena, either from thrombi formation on the tumor’s surface or by detachment of tumor fragments into the systemic circulation. These emboli may result in cerebral infarctions, and there is growing interest in whether myxomas could also trigger inflammatory responses affecting cerebral blood vessels, resembling aspects of vasculitis [5].

Diagnosing cardiac myxomas can be challenging. TTE is the primary imaging modality due to its high sensitivity, allowing the assessment of the tumor’s characteristics (morphology, size, mobility, and attachment site). Doppler evaluation assesses the hemodynamic consequences of the mass. Complementary TOE provides a more detailed evaluation, and cardiac MRI can be useful in establishing a differential diagnosis with other cardiac masses [5].

Currently, there is no effective medical treatment to stop myxomas growth, and surgical removal is necessary to prevent life-threatening complications [6].

The timing of surgery is controversial in patients with recent neurological ischemic injuries, due to the risk of bleeding conversion during cardiopulmonary bypass [7].

Surgical resection is optimized by inducing cardioplegic arrest to provide a motionless operative field and realize a piecemeal removal. Aortic cross-clamping helps minimize perioperative tumor dislodgement and massive embolization, particularly with papillary and friable myxomas.

The usual approach is via left atrial incision, posterior to interatrial groove to explore the tumor. In cases of giant myxomas or restricted left atrial access, the approach can be via right atriotomy. There is controversy regarding radical resection with excision of a full-thickness portion of the interatrial septum versus conservative excision, especially with sporadic myxomas [8].

Surgical excision has an excellent long-term prognosis. The overall risk of recurrence is 22% for Carney complex myxomas, 12% for familial myxomas, and only 1% to 3% for sporadic cases [9].

Although surgical resection is curative with a low recurrence rate, patients should undergo regular follow-up with serial TTE [10].

## Conclusion

We presented an interesting case featuring an uncommon and misleading neurological presentation of cardiac myxoma.

This case underscores the critical need for heightened clinical suspicion and thorough diagnostic evaluation to achieve early recognition among a broad range of differential diagnoses.

Recognizing and promptly treating cardiac myxomas is imperative to prevent life-threatening complications.

## Data Availability

No datasets were generated or analyzed during the current study.

## Abbreviations

CT: Computed tomography
MRA: Magnetic resonance angiography
EKG: Electrocardiogram
TTE: Transthoracic echocardiography
TOE: Trans esophageal echocardiography
ICU: Intensive care unit
IL-6: Interleukin-6
